# Intersection of Poverty and Rurality for Early-Onset Colorectal Cancer Survival

**DOI:** 10.1001/jamanetworkopen.2024.30615

**Published:** 2024-08-28

**Authors:** Meng-Han Tsai, Steven S. Coughlin, Jorge Cortes, Caroline A. Thompson

**Affiliations:** 1 Georgia Prevention Institute, Augusta University, Augusta; 2 Cancer Prevention, Control, and Population Health Program, Georgia Cancer Center, Augusta University, Augusta; 3 Department of Biostatistics, Data Science, and Epidemiology, School of Public Health, Augusta University, Augusta, Georgia; 4 Georgia Cancer Center, Augusta University, Augusta; 5 Department of Epidemiology, Gillings School of Global Public Health, University of North Carolina at Chapel Hill; 6 Lineberger Comprehensive Cancer, University of North Carolina at Chapel Hill

## Abstract

This cross-sectional study examines poverty, rurality, and the intersection of persistent poverty and rurality on early-onset colorectal cancer survival among adults aged 18 to 49 years.

## Introduction

Early-onset colorectal cancer (EO-CRC; defined as colorectal cancer [CRC] diagnosed in individuals younger than 50 years) has increased recently in the US.^1^ Evidence has shown that patients living in impoverished and rural areas have an increased risk of CRC death,^2,3^ but this has not been examined for EO-CRC. Thus, we examined the associations of EO-CRC mortality with persistent poverty, rurality, and the intersection of persistent poverty and rurality overall and within age groups.

## Methods

Data extracted for this study were publicly available and deidentified, and thus considered exempt from review by the institutional review board at Augusta University, and informed consent was not required. This study followed the Strengthening the Reporting of Observational Studies in Epidemiology (STROBE) reporting guideline. Additional information can be found in the eAppendix in Supplement 1.

We analyzed the 2006 to 2015 Incidence Data with Census Tract Attributes from Surveillance, Epidemiology, and End Results (SEER) Program. Cause-specific survival for CRC was the outcome of interest; deaths from other causes were censored. Census tract persistent poverty (yes or no) and rurality (yes or no) were our exposures of interest, with stratification by SEER age group (20 to 29 years, 30 to 39 years, and 40 to 49 years). Census tract persistent poverty was previously defined as 20% or more of the population living below the poverty level for a period spanning approximately 30 years.^4^ Rurality was defined by US Department of Agriculture Rural Urban Commuting Area codes, with 4.0, 4.2, 5.0, 5.2, 6.0 to 7.0, 7.2 to 8.0, 8.2 to 10.0, and 10.2 to 10.6 considered as rural.^4^ The Kaplan-Meier method with log-rank test and multivariable confounders (ie, patient demographics, year of diagnosis, tumor characteristics, initial treatment modality) adjusted Cox proportional hazards regression models were performed. Data were analyzed using SAS version 9.4 (SAS Software). Statistical significance was a 2-sided *P* < .05. Data were analyzed from February 15 to February 28, 2024.

## Results

Among 58 200 patients with EO-CRC, 42 694 were aged 40 to 49 years (73%) and 30 580 (53%) were male. Among patients included in this study, 1346 patients (21%) lived in rural areas with persistent poverty. Overall, 5-year survival was highest for those living in nonpoverty and nonrural areas (46 566 [72%]) and lowest for those living in poverty areas regardless of rurality (6480 [67%]), with some variation by age group (eg, survival was 64% for those aged 20 to 29 years living in impoverished rural areas). In multivariable analysis, patients with EO-CRC who lived in rural areas alone had 1.1-fold to 1.4-fold increased risk of CRC death compared with those living in nonrural areas (20 to 29 years: hazard ratio [HR], 1.35; 95% CI, 1.06-1.71; 30 to 39 years: HR, 1.26; 95% CI, 1.13-1.41; 40 to 49 years: HR, 1.12; 95% CI, 1.06-1.19). Those living in both poverty and rural areas had a 1.1-fold to 1.5-fold increased risk of CRC death compared with those living in nonrural areas (overall: HR, 1.29; 95% CI, 1.18-1.42) with notably high estimates for that aged 30 to 39 years (HR, 1.51; 95% CI, 1.22-1.88) (Table).

**Table.** Persistent Poverty and Rurality and the Risk of EO-CRC Death by Age at Diagnosis

**Age group**	Rurality										HR (95% CI) for rurality within strata of poverty^e^
	No					Yes					
	No. (%)^a^			HR (95% CI)^b^^,^^c^	HR for poverty within strata of rurality (95% CI)^f^	No. (%)^a^			HR (95% CI)^b^^,^^c^	HR for poverty within strata of rurality (95% CI)^d^	
	Patients	5-y CRC survival				Patients	5-y CRC survival				
		No	Yes				No	Yes			
**All age groups**											
No persistent poverty (n = 51 720)	46 566 (90.0)	13 128 (28.2)	33 438 (71.8)	1 [Reference]	1.08 (1.02-1.14)	5154 (10.0)	1578 (30.6)	3576 (69.4)	1.16 (1.11-1.22)	1.11 (1-1.24)	1.16 (1.1-1.22)
Persistent poverty (n = 6480)	5134 (79.2)	1720 (33.5)	3414 (66.5)	1.08 (1.02-1.14)		1346 (20.8)	446 (33.1)	900 (66.9)	1.29 (1.18-1.42)		1.2 (1.08-1.33)
**20 to 29 years**											
No (n = 2859)	2578 (90.2)	658 (25.5)	1920 (74.5)	1 [Reference]	1.07 (0.87-1.31)	281 (9.8)	80 (28.5)	201 (71.5)	1.35 (1.06-1.71)	0.82 (0.53-1.28)	1.35 (1.06-1.71)
Yes (n = 442)	367 (83.0)	118 (32.2)	249 (67.8)	1.07 (0.87-1.31)		75 (17.0)	27 (36.0)	48 (64.0)	1.11 (0.75-1.63)		1.03 (0.67-1.58)
**30 to 39 years**											
No (n = 10 875)	9857 (90.6)	2715 (27.5)	7142 (72.5)	1 [Reference]	1.03 (0.92-1.16)	1018 (9.4)	324 (31.8)	694 (68.2)	1.26 (1.12-1.42)	1.2 (0.95-1.53)	1.26 (1.12-1.42)
Yes (n = 1330)	1065 (80.0)	357 (33.5)	708 (66.5)	1.03 (0.92-1.16)		265 (19.9)	85 (32.1)	180 (67.9)	1.51 (1.22-1.88)		1.47 (1.16-1.87)
**40 to 49 years**											
No (n = 37 986)	34 131 (89.9)	9755 (28.6)	24 376 (71.4)	1 [Reference]	1.09 (1.02-1.16)	3855 (10.1)	1174 (30.5)	2681 (69.6)	1.12 (1.06-1.19)	1.13 (0.99-1.27)	1.12 (1.06-1.19)
Yes (n = 4708)	3702 (78.6)	1245 (33.6)	2457 (66.4)	1.09 (1.02-1.16)		1006 (21.4)	334 (33.2)	672 (66.8)	1.26 (1.13-1.41)		1.16 (1.03-1.31)

Abbreviations: CRC, colorectal cancer; EO-CRC, early-onset colorectal cancer; HR, hazard ratio.

^a^ Row percentages were used.

^b^ Models were adjusted for patient demographics (gender, race, marital status at diagnosis), year of diagnosis, tumor characteristics (tumor grade, tumor stage, primary site), and initial treatment modality (surgery, radiation, chemotherapy).

^c^ Reference group is rural = No; poverty = No.

^d^ Reference group is rural = No.

^e^ Reference group poverty = No.

## Discussion

In this study, patients with EO-CRC living in rural areas had lower 5-year survival rates than their urban dwelling counterparts. While it was not observed consistently for all age groups, persistent poverty in these rural areas may compound this association. For example, 30 to 39-year-olds living in rural and impoverished areas had a 50% greater risk of CRC death than those living in other areas. This intersecting association with risk of CRC death was also more pronounced for patients with EO-CRC by 30% compared with 19% in average-onset CRC.

Potential explanations for this association include patients with EO-CRC who lived in rural areas may have been diagnosed with later stage disease more frequently,^5^ underinsurance among younger adults, especially in nonexpanded Medicaid states with high rurality, or lower quality treatment received by patients in rural or impoverished areas.^5^ Other considerations may include higher levels of comorbidities (eg, obesity) among younger adults who lived in rural areas, which could complicate the likelihood of treatment success.^6^

EO-CRC is a rising concern nationwide but underresourced areas face unique challenges. Our results can be used to inform health system policies for ongoing investments in cancer diagnosis and treatment resources in rural or impoverished areas for younger CRC patients and their communities. These may include education programs to encourage healthy lifestyles, and symptom awareness campaigns tailored to young adults to improve earlier diagnosis. This study is limited by the inability to adjust for other important confounders, such as lifestyle factors, comorbidities, and structural barriers, which are not generally available in SEER program.

## Notes

Supplement 1. **eAppendix.** Study Design

Supplement 2.
Data Sharing Statement
