# Supplementary material for: Intersection of Poverty and Rurality for Early-Onset Colorectal Cancer Survival
Source: JAMA Netw Open. 2024 Aug 28;7(8):e2430615. doi: 10.1001/jamanetworkopen.2024.30615 (PMC11358859; doi:10.1001/jamanetworkopen.2024.30615)
Supplement: Supplement 1. — eAppendix. Study Design [file jamanetwopen-e2430615-s001.pdf]

Supplemental Online Content

Tsai MH, Coughlin SS, Cortes J, Thompson CA. Poverty, rurality, and early-onset colorectal cancer survival. *JAMA Netw Open*. 2024;7(8):e2430615. doi:10.1001/jamanetworkopen.2024.30615

eAppendix. Study Design

This supplemental material has been provided by the authors to give readers additional information about their work.

© 2024 Tsai MH et al. *JAMA Network Open*.

eAppendix. Study Design

Study design for eligible sample: First, patients diagnosed with colorectal cancer (CRC) defined by the SEER Site Recode ICD-O-3/WHO 2008 definition of colon cancer (C180–C189), rectosigmoid junction cancer (C199), and rectal cancer (C209) were used to select CRC patients through SEER* stat. Furthermore, cause-specific death for CRC was defined by using SEER causes-specific death classification.

© 2024 Tsai MH et al. *JAMA Network Open.*
