# Supplementary material for: Intersection of Poverty and Rurality for Early-Onset Colorectal Cancer Survival
Source: JAMA Netw Open. 2024 Aug 28;7(8):e2430615. doi: 10.1001/jamanetworkopen.2024.30615 (PMC11358859; doi:10.1001/jamanetworkopen.2024.30615)
Supplement: Supplement 2. — Data Sharing Statement [file jamanetwopen-e2430615-s002.pdf]

Data Sharing Statement

Data
Data available: Yes
Data types: Other (please specify)
Additional Information: The datasets generated during the current study are available in the Surveillance, Epidemiology, and End Results Program (https://seer.cancer.gov/).
How to access data: The datasets generated during the current study are available in the Surveillance, Epidemiology, and End Results Program (https://seer.cancer.gov/)
When available: With publication

Supporting Documents
Document types: None

Additional Information
Who can access the data: Anyone requesting the data
Types of analyses: for any purpose
Mechanisms of data availability: without investigator support
